# Lineage Analysis of *Drosophila* Lateral Antennal Lobe Neurons Reveals Notch-Dependent Binary Temporal Fate Decisions

**DOI:** 10.1371/journal.pbio.1001425

**Published:** 2012-11-20

**Authors:** Suewei Lin, Chih-Fei Kao, Hung-Hsiang Yu, Yaling Huang, Tzumin Lee

**Affiliations:** 1 Janelia Farm Research Campus, Howard Hughes Medical Institute, Ashburn, Virginia, United States of America; 2 Department of Neurobiology, University of Massachusetts Medical School, Worcester, Massachusetts, United States of America; New York University, United States of America

## Abstract

Binary cell fate decisions allow the production of distinct sister neurons from an intermediate precursor. Neurons are further diversified based on the birth order of intermediate precursors. Here we examined the interplay between binary cell fate and birth-order-dependent temporal fate in the *Drosophila* lateral antennal lobe (lAL) neuronal lineage. Single-cell mapping of the lAL lineage by twin-spot mosaic analysis with repressible cell markers (ts-MARCM) revealed that projection neurons (PNs) and local interneurons (LNs) are made in pairs through binary fate decisions. Forty-five types of PNs innervating distinct brain regions arise in a stereotyped sequence; however, the PNs with similar morphologies are not necessarily born in a contiguous window. The LNs are morphologically less diverse than the PNs, and the sequential morphogenetic changes in the two pairs occur independently. Sanpodo-dependent Notch activity promotes and patterns the LN fates. By contrast, Notch diversifies PN temporal fates in a Sanpodo-dispensable manner. These pleiotropic Notch actions underlie the differential temporal fate specification of twin neurons produced by common precursors within a lineage, possibly by modulating postmitotic neurons' responses to Notch-independent transcriptional cascades.

## Abstract 2

A high-resolution neuronal lineage analysis in the *Drosophila* antennal lobe reveals the complexity of lineage development and Notch signaling in cell fate specification.

## Author Summary

The *Drosophila* brain develops from a limited number of neural stem cells that produce a series of ganglion mother cells (GMCs) that divide once to produce a pair of neurons in a defined order, termed a neuronal lineage. Here, we provide a detailed lineage map for the neurons derived from the *Drosophila* lateral antennal lobe (lAL) neuroblast. The lAL lineage consists of two distinct hemilineages, generated through differential Notch signaling in the two GMC daughters, to produce one projection neuron (PN) paired with a local interneuron (LN). Both hemilineages yield distinct cell types in the same sequence, although the temporal identity (birth-order-dependent fate) changes are regulated independently between projection neurons and local interneurons, such that a series of analogous local interneurons may co-derive with different projection neurons and vice versa. We also find that Notch signaling can transform a class of nonantennal lobe projection neurons into antennal lobe projection neurons. These findings suggest that Notch signaling not only modulates temporal fate but itself plays a role in the distinction of antennal lobe versus nonantennal lobe neurons.

## Introduction

The computing power of a brain is rooted in its complex neural network, composed of numerous types of neurons. Understanding how diverse neurons are specified is fundamental for elucidating how such an intricate organ develops and evolves from simple to higher organisms. *Drosophila melanogaster* has a relatively tractable neural development, study of which has revealed multiple mechanisms that act in sequence to diversify neuron fates [1]. To determine the interplay among serial fating mechanisms is critical for unraveling how the large repertoire of neural fates can be reliably established to make a functional brain.

Neurons in the *Drosophila* central nervous system (CNS) arise from a stereotyped set of neural progenitors called neuroblasts (NBs) [2],[3]. Each NB generates a lineage of neurons through multiple rounds of self-renewing asymmetric cell divisions. In most divisions, one NB deposits a ganglion mother cell (GMC) that divides once to produce two neurons [4],[5]. Three known mechanisms underlie neuronal diversification through the protracted process of neurogenesis. (1) The acquisition of lineage identity by each NB occurs during early spatial patterning and governs the neural types it produces [6]. (2) The specification of temporal identity within a given lineage underlies the orderly derivation of distinct neurons from a common progenitor [7],[8]. (3) The binary cell fate decision distinguishes fate between sister neurons made by a single GMC [9]–[14].

Although much has been learned about each of the neuronal diversification processes, scientists have just begun to elucidate how these serial fating mechanisms are integrated to determine a neuron's terminal fate. A combinatorial expression of various transcription factors may confer lineage identity based on where the NBs originate in early embryos [6],[15]–[21]. By contrast, a generic temporal fating mechanism has been shown to govern birth order/time-dependent neuron fate specification in diverse neuronal lineages [7],[8]. It involves a series of transcription factors that express in sequence in the NBs. Each of these transcription factors dictates the temporal identity of the neurons born during the time of its expression [22]. However, distinct lineages show different lineage-characteristic temporal identity profiles, arguing for a role of lineage identity in patterning the expression of temporal factors. In fact, recent studies on the *Drosophila* embryonic NB lineage 5–6 have demonstrated that lineage determinants and temporal fating factors do not simply work additively to specify a final neuron fate. Instead, lineage identity genes may refine neuronal temporal fates by subdividing the temporal window defined by a single temporal identity factor into multiple subdomains with distinct transcriptional outputs [23]. As to the binary cell fate decision in postmitotic neurons, it remains unclear how the transition of the temporal code of the NB and GMC precursors is differentially read out based on Notch activity. Do sister neurons made by the same set of GMCs in a given lineage alter temporal identity simultaneously? If not, what mechanisms underlie the differential patterning of temporal cell fates between the Notch-on neurons and their Notch-off sibs?

Notch/Numb has been shown to specify A/B binary cell fates of twin neurons derived from a GMC [13],[14]. Such binary fate decision underlies the initial production of two distinct sets of progeny in most, if not all, *Drosophila* neuronal lineages. However, many CNS lineages exist as a lone hemilineage because one entire hemilineage has undergone premature cell death [24]–[26]. For instance, two of the three antennal lobe (AL) lineages, which make projection neurons (PNs) connecting the AL to the lateral horn (LH), yield only one viable neuron from each GMC. Notably, Notch-off specifies the PN fates and Notch-on promotes apoptosis in the anterodorsal PN (adPN) lineage but vice versa in the ventral PN (vPN) lineage [24]. Mapping (delineating the serially derived neurons based on the GMC birth order) the adPN lineage has revealed 40 types of AL PNs that arise in an invariant sequence from the progenitor of the lineage [27]. Unfortunately, it is not possible to discern birth time/order-dependent fate changes among their apoptotic sibs, preventing comparative analysis of temporal fate transitions between sister hemilineages. By contrast, the lAL lineage produces PNs as well as AL local interneurons (LNs), which can be interconverted by manipulating Notch activities [24],[28]. Identifying each PN and LN and determining their twin relationship in the lAL lineage should allow a close examination of neuron fate specification based on the interplay between temporal identity and binary fate decision.

Here we determined the twin neurons made by each GMC of the larval lAL lineage, using twin-spot mosaic analysis with repressible cell markers (ts-MARCM) that permits labeling of sister clones (e.g., twin neurons) derived from a common precursor (e.g., a GMC) in distinct colors [26]. We demonstrated that the lAL lineage consists of two distinct hemilineages that yield a PN and LN pair from each GMC at early times and a single PN at the end of the lineage. Stereotyped PN and LN twin clones were consistently observed at specific time points. Notably, PNs exhibit higher morphological diversity and thus alter temporal fates that govern morphogenesis in a faster tempo than their LN sibs. Additional lines of evidence indicate that the PN and LN offspring of the lAL lineage are differentially patterned with respect to their temporal identity. Interestingly, knocking out Sanpodo (Spdo), a positive regulator of Notch, from the lAL NB led to duplication of the entire complement of PNs at the expense of less dynamic LNs. This implies that twin neurons are born with equivalent temporal codes, which may specify different temporal fates depending on Notch activities. We further uncovered a *spdo*-independent role of Notch in specifying a set of temporal fates in the PN hemilineage. Despite the complex binary and temporal fate transformations, *Notch* mutant clones maintained the normal dynamic expressions of Chronologically inappropriate morphogenesis (Chinmo) [29],[30] and Broad complex (Br-C) [31] during larval development. Although Notch did not regulate *chinmo* expression, loss of Chinmo affected PN and LN temporal fates in hemilineage-dependent manners, arguing that Notch acts downstream of temporal fating factors to modulate neuronal temporal fates. Taken together, we established the *Drosophila* lAL lineage as a model system for studying the origin-dependent neuron fate specification and demonstrated that Notch not only underlies binary cell fate decision but also determines temporal fates in both Notch-high and Notch-low sister neurons.

## Results

### Projection Neurons and Local Interneurons Are Made in Pairs from Common Ganglion Mother Cells in the Lateral Antennal Lobe Lineage

**Figure 1:**
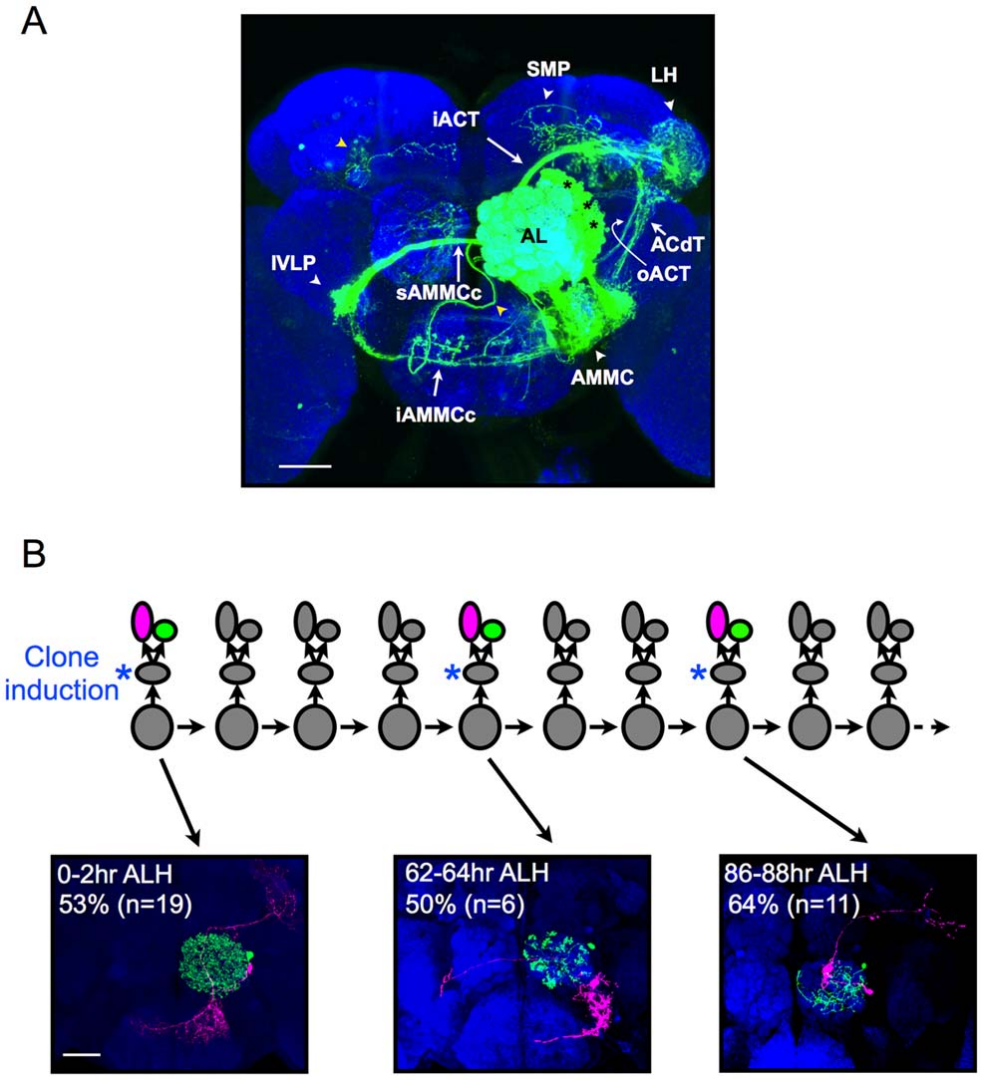
lAL neurons are born as LN/PN pairs. (A) A lAL NB clone (green), generated upon larval hatching and labeled by *nSyb-GAL4*, a pan-neural driver, in an adult brain. The brain was counterstained with nc82 mAb to reveal synaptic neuropiles (blue). The brain regions densely innervated by the lAL neurons are indicated by white arrowheads. Two background processes are indicated by yellow arrowheads. AL, antennal lobe; IVLP, inferior ventrolateral protocerebrum; AMMC, antennal mechanosensory and motor center; LH, lateral horn; SMP, superior medial protocerebrum. The major tracts are indicated by arrows. iACT, inner antennocerebral tract; oACT, outer antennocerebral tract; ACdT, antennocerebral descending tract; sAMMCc, superior AMMC commissure; iAMMCc, inferior AMMC commissure. The stars mark the cell bodies of the lAL neurons. Scale bar: 40 µm. (B) The illustration shows the proliferation mode of the lAL NB/GMCs and how ts-MARCM labels the twin-cells born at different times. The ts-MARCM was induced in the dividing GMCs by mild heat shock; the two daughter cells from each of the dividing GMCs were then labeled by different fluorescent proteins. The images at the bottom are examples of the ts-MARCM clones induced at 0–2 h, 62–64 h, and 86–88 h ALH. The LNs and PNs were pseudocolored in green and magenta, respectively. Note the sister cells from a GMC were one PN and one LN, and the neurons with different birthdates showed different morphologies. Scale bar: 40 µm. The background clones in these images were masked as described in Materials and Methods.

The lateral antennal lobe (lAL) lineage yields about 200 neurons during larval neurogenesis [32]. Labeling the lAL progeny by conventional mosaic analysis with a repressible cell marker (MARCM) [33] using a pan-neural *nSyb-GAL4* revealed neuronal cell bodies packed along the lateral border of the AL. They elaborate densely in the antennal lobe (AL) and the neighboring antennal mechanosensory and motor center (AMMC) (Figure 1A) and further innervate the inferior ventrolateral protocerebrum (IVLP), lateral horn (LH), superior medial protocerebrum (SMP), and some other brain regions (Figure 1A). It is not possible to determine the detailed “projectome” among the targets without single-neuron labeling.

To reveal single-cell morphology and simultaneously determine the neuron birth order, we “sequenced” the larval lAL lineage using ts-MARCM [26] with *nSyb-GAL4*. We determined ganglion mother cell (GMC) progeny born in 2-h windows throughout larval development (see Materials and Methods). We identified lAL GMC clones based on cell body positions and neurite trajectory patterns that match the lAL NB clones generated at various time points (unpublished data). Except near the lineage end (see below), both daughter neurons derived from each lAL GMC survived into the adult stage. Notably, one projection neuron (PN) consistently paired with one local interneuron (LN). They exist as twin clones when differentially labeled by ts-MARCM (Figure 1B). This confirms the previous hypothesis that the lAL lineage is composed of one PN hemilineage and one LN hemilineage [24],[28],[32]. Moreover, we obtained neuron pairs with distinct characteristic morphologies following clone induction at different developmental times, indicating that the birth order of GMCs has governed neuronal diversification in the protracted lAL lineage (Figure 1B; see below).

### The Lateral Antennal Lobe Projection Neurons Innervate Brain Regions That Are Involved in Different Sensory Modalities

**Figure 2:**
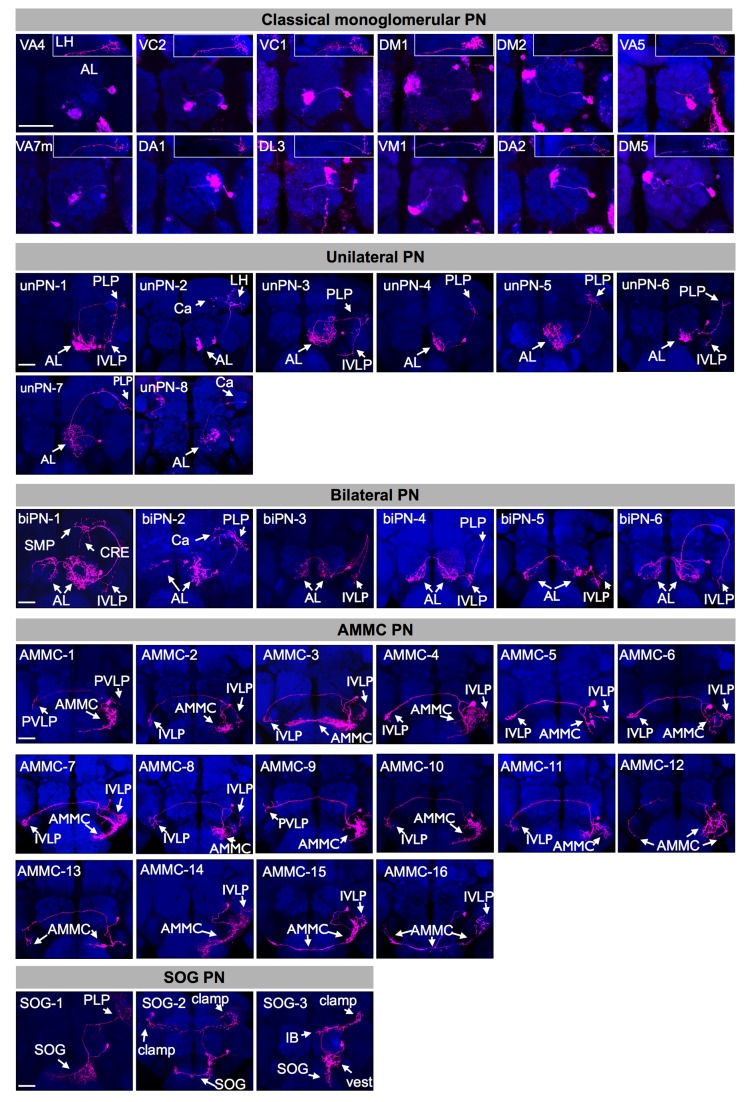
The lAL PNs can be grouped into five classes based on neuron morphology. Single PNs labeled by ts-MARCM with *nSyb-GAL4* (magenta). Their LN sibs are not shown. Brains were co-stained with nc82 mAb (blue). Based on their morphology, the lAL PNs are grouped into classical monoglomerular PN (mPN), unilateral PN (unPN), bilateral PN (biPN), antennal mechanosensory and motor center (AMMC) PN, or suboesophageal ganglion (SOG) PN. The dendrites of each mPN innervate a single glomerulus in AL and its axon project to mushroom body (MB) and lateral horn (LH) through inner antennocerebral tract (iACT). The unPNs and biPNs also have proximal innervations in AL, but unPNs innervate ipsilateral AL only and biPNs innervate both ipsilateral and contralateral ALs. Different from mPNs that project axons exclusively to MB and LH, unPNs and biPNs have distal projections targeting many other brain regions, often not through iACT. The AMMC and SOG PNs do not innervate AL but instead innervate AMMC and SOG, respectively. The brain regions innervated by each type of the lAL neurons are marked (arrows). The 12 types of mPN are named according to the glomeruli they innervate in AL, and their AL and lateral horn (LH) innervation are shown separately. Scale bar: 40 µm. Except for the AL region of mPNs, the background clones in these images were masked as described in Materials and Methods. AL, antennal lobe; cAL, contralateral AL; PLP, posteriorlateral protocerebrum; PVLP, posterior ventrolateral protocerebrum; cPVLP, contralateral posterior ventrolateral protocerebrum; SOG, suboesophageal ganglion; IB, inferior bridge; Ca, mushroom body calyx; LH, lateral horn; AMMC, antennal mechanosensory and motor center; cAMMC, contralateral antennal mechanosensory and motor center; IVLP, inferior ventrolateral protocerebrum; cIVLP, contralateral inferior ventrolateral protocerebrum; SMP, superior medial protocerebrum; CRE, crepine.

The lAL PNs identified by ts-MARCM can be categorized into five classes based on their morphology: monoglomerular PN (mPN), unilateral PN (unPN), bilateral PN (biPN), AMMC PN, and suboesophageal ganglion (SOG) PN (Figure 2). mPNs connect a single AL glomerulus to the mushroom body (MB) calyx and LH through the inner antennocerebral tract (iACT; Figure 1A) [34],[35]. The mPNs target the VA4, VC2, VC1, DM1, DM2, VA5, VA7m, DA1, DL3, VM1, DA2, or DM5 AL glomerulus (Figure 2) and have been determined previously based on the *GAL4-GH146* marker [36],[37]. The lack of additional mPNs using the more broadly expressed nSyb marker suggests this set was already complete. Unlike mPNs, unPNs and biPNs connect the AL(s) to various brain regions not yet implicated in olfaction, which include the posteriorlateral protocerebrum (PLP), inferior ventrolateral protocerebrum (IVLP), and superior medial protocerebrum (SMP). unPNs restrict their proximal elaborations to the ipsilateral AL, whereas biPNs show bilateral AL elaborations. Eight types of unPNs and six types of biPNs can be further distinguished based on (1) AL innervation patterns, (2) neurite trajectories, and (3) distal targets (Figure 2; see Table S1 for details).

In addition to the AL PNs, we obtained 16 types of AMMC neurons and three types of SOG neurons that may account for the AMMC and SOG elaborations seen in the lAL NB clones (Figure 2, compared to Figure 1A). Most of the AMMC neurons acquire some bilateral elaborations across the brain midline. AMMC-1 to -11 connect the ipsilateral AMMC to the ipsilateral as well as contralateral IVLPs or posterior ventrolateral protocerebrum (PVLP) (AMMC-1 to -8), or only to the contralateral IVLP/PVLP (AMMC-9 to -11). AMMC-12 and -13 elaborate exclusively within the AMMC and wire the paired AMMC structures together. AMMC-14 to -16 show dendrite-like processes in the IVLP and axon-like projections in the AMMC, but AMMC-14 only targets the ipsilateral AMMC whereas AMMC-15 and -16 innervate both ipsilateral and contralateral AMMCs. Finally, the three types of SOG PNs show unique characteristic patterns of proximal elaboration in the SOG and further target distinct brain regions, including the PLP (SOG-1), the contralateral and ipsilateral clamp surrounding the MB peduncles (SOG-2), and the ipsilateral clamp and inferior bridge (IB) (SOG-3). Some proximal neurites of SOG-3 further innervate the vest, which is posterior to the AL. Please refer to Table S1 for more detailed description of these stereotyped AMMC and SOG neurons.

In sum, the lAL NB yields not only AL PNs but also AMMC and SOG neurons, which may contribute to distinct neural circuits (see Discussion). Does the GMC birth order guide the derivation of these multiclass neurons one group by another along the Notch-off hemilineage of the complex lAL pedigree [24],[28]?

### Orderly, But Not Class-By-Class, Production of Distinct Lateral Antennal Lobe Projection Neurons

**Figure 3:**
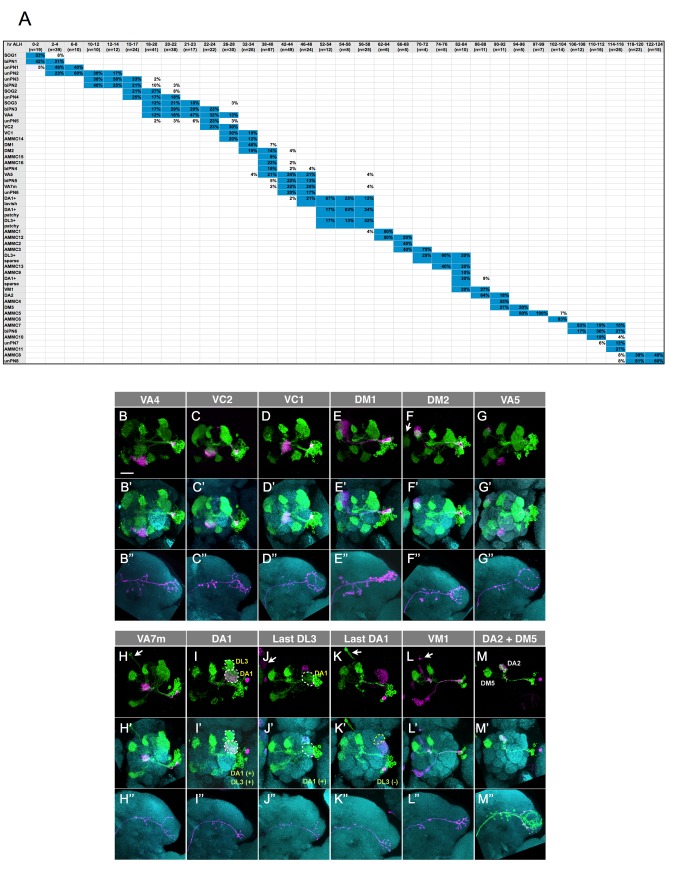
The lAL PNs are produced in a specific birth order. (A) The table shows the percentage (numbers in the table body) of clones labeling individual PN types (listed in the header column) in all the clones induced at a specific developmental time (indicated as hours after larval hatching [h ALH] in the header row). Only the boxes with the percentage of clones equal to or above 10% are filled with blue. The PNs paired with different types of LNs are separated and put in different rows; for example, “DA1+lavish (LN)” and “DA1+patchy (LN)” are put in separate rows. (B–M″) The *GAL4-GH146*-labeled ts-MARCM neuroblast (NB) clones of the lAL lineage induced at different developmental times to reveal the birth order of the classical monoglomerular projection neurons (mPNs). The clones are arranged by their birth order from early to late. The single-cell side of the ts-MARCM clone is pseudocolored in magenta, and the NB side of the clone is pseudocolored in green. Brains were co-labeled with nc82 mAb (cyan; B′–M″). The dendritic elaboration in AL (B–M′) and the axonal terminals in LH (B″–M″) are shown separately. The background clones are indicated by arrows. Note the sequential loss of earlier born neurons from the green NB clones. Scale bar: 20 µm.

The twin single-cell clones collected for this study were induced in discrete 2-h windows to sample neurons born at different times from larval hatching to puparium formation. Notably, distinct lAL PNs were preferentially hit at different developmental times. To deduce their possible birth order, we attempted to arrange the identified lAL PN types chronologically based on when their precursors are susceptible to mitotic recombination. We first determined the primary window(s) of susceptibility for each lAL PN type (shaded boxes in Figure 3A; boxes that account for less than 10% or less of the hits at the respective timing of clone induction are not shaded, except for rarely hit neuron types, including AMMC-9, and AMMC-15). All but two of the 45 identifiable PN types show a single narrow window of susceptibility that staggers in partially overlapping manners along the ∼120 h of larval development. A tentative PN birth order can then be deduced based on the starts and/or ends of the susceptible windows as well as their prime times of appearance (Figure 3A).

We also determined the sequence of birth for the mPNs through analysis of *GAL4-GH146*-labeled NB clones (Figure 3B–M″). We witnessed a sequential loss of the 12 glomerular targets from the NB clones of reducing sizes. As to their paired GMC clones, we observed the serial appearance of the VA4, VC2, VC1, DM1, DM2, VA5, and VA7m mPNs as they sequentially disappeared from the NB clones of reducing sizes (Figure 3B–H″). Then some DA1 mPNs were hit before the birth of DL3 mPNs, and additional DA1 mPNs arose later with the NB clones lacking DL3 mPNs (Figure 3I–K″). The remaining VM1, DA2, and DM5 mPNs then followed in the same sequence as they disappeared from the NB clones (Figure 3L–M″). In addition, certain NB clones apparently paired with GH146-negative progeny and existed alone when labeled with *GAL4-GH146* (unpublished data). Ignoring those gaps, we derived the following birth sequence for the 12 types of lAL mPNs: VA4-VC2-VC1-DM1-DM2-VA5-VA7m-DA1-DL3-DA1-VM1-DA2-DM4. The same birth order was obtained from the analysis of *nSyb-GAL4*-labeled single-cell clones (Figure 3A). Notably, mapping the lAL lineage using a ubiquitous driver, like *nSyb-GAL4*, and through analysis of numerous serially derived single-cell clones (Figure 3A), further allowed us to (1) fill the gaps occupied by GH146-negative PNs, (2) resolve the mixing of DA1/DL3 mPNs, and (3) uncover the paired LNs (see below).

The complete birth order of larval-derived lAL PNs unveils several interesting points. First, distinct PNs are born in an invariant sequence. Second, different PN classes are born in an intercalated sequence with analogous PN types arising in separate windows. For example, the 12 mPN types derive in nine blocks that span nearly two-thirds of the larval development. During the same period of time, 14 other PN types, including 10 types of AMMC neurons, are made. Six additional AMMC types plus three types of atypical AL PNs are derived afterwards. In contrast with the late AMMC siblings, the majority of atypical AL PNs and all the SOG neurons are born prior to the mPN-producing windows. Third, the apparently arbitrary birth order is further complicated by the recurrent production of DA1 and DL3 mPNs. They are first born from 46 to 58 h after larval hatching and are also generated roughly 12 h later (Figure 3A). DA1 mPNs precedes DL3 mPNs during their initial contiguous production. By contrast, DL3 mPNs arise before DA1 mPNs in their second round of birth that is further separated by the production of two types of AMMC neurons. The early-born DA1/DL3 and later-derived DA1/DL3 mPNs are morphologically indistinguishable and are both positive for *GAL4-GH146*. Nonetheless, the DA1/DL3 mPNs born at different times pair with distinct LNs, and the early-born DA1 mPNs can be further divided into two groups based on their paired LNs (Figure 3A; see below).

Taken together, the lAL NB makes distinct PNs of diverse classes in a fixed arbitrary sequence. Neurons acquire specific fates based on their birth order, but the actual sequence of production reveals no obvious logic behind their stereotyped temporal deployment. Analogous neurons can arise at different times across the protracted lineage. Moreover, identical neurons can be born consistently in two waves. To uncover the genes that determine specific neuron classes versus the fates within a class will be critical for elucidating the molecular mechanisms underlying such orderly, but not class-by-class, production of distinct neuronal siblings.

### Diverse Local Interneurons Pair with Distinct Projection Neurons to Account for 48 Serially Derived Neuron Pairs

**Figure 4:**
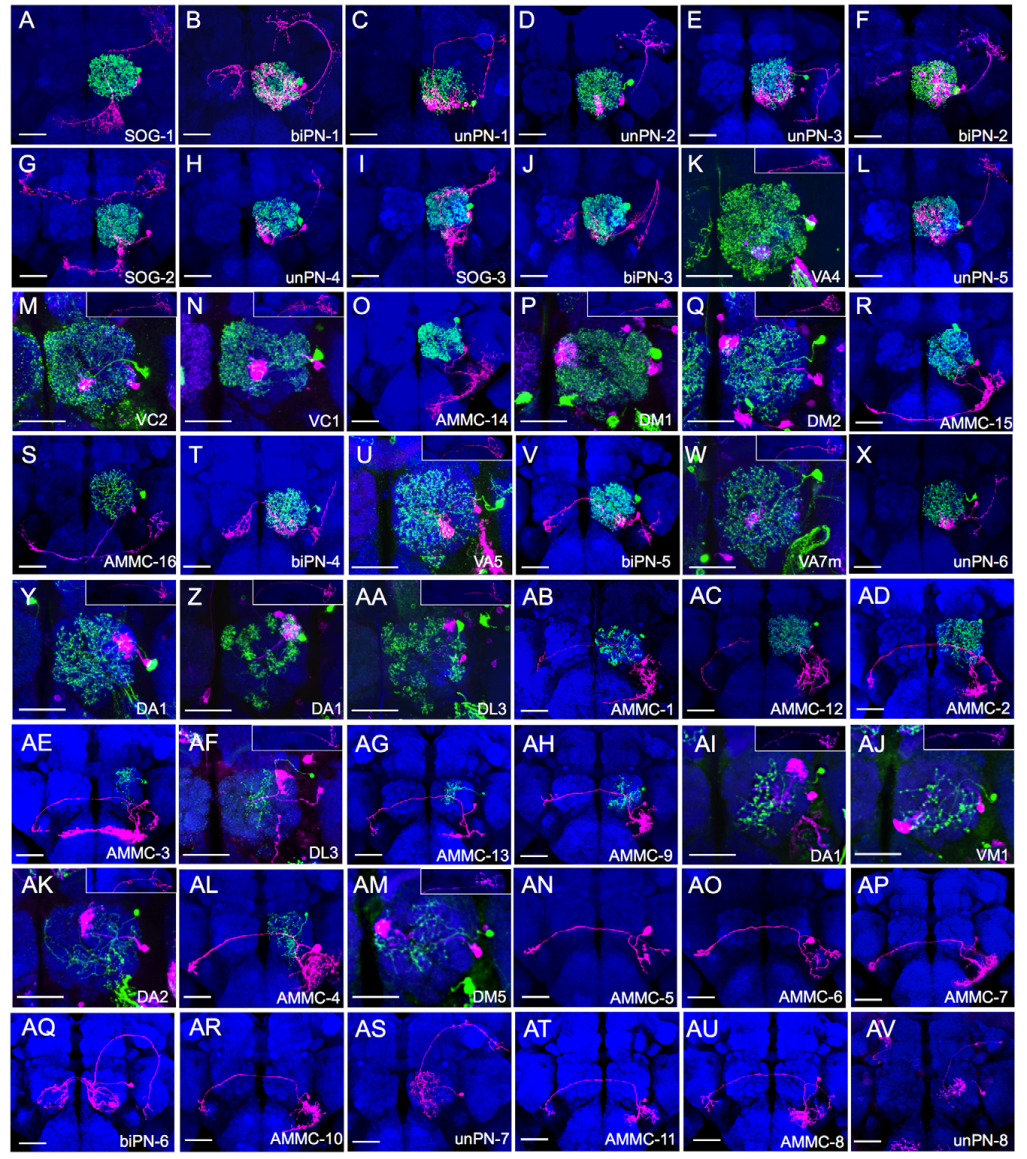
The larval lAL lineage yields PNs and LNs in pairs except near the lineage end. The lAL PN/LN pairs (A–AM) and the lone lAL PNs (AN–AV) arranged by their birth order from early to late. The neurons were labeled by ts-MARCM with *nSyb-GAL4*; LNs were pseudocolored in green and PNs were pseudocolored in magenta. Brains were counterstained with nc82 mAb (blue) to reveal synaptic neuropil. The axonal terminal in the lateral horn and the dendritic elaboration in the antennal lobe are shown separately for the 12 types of classical mPNs (K,M,N,P,Q,U,W,Y,Z,AA,AF,AI,AJ,AK,AM). Scale bar: 40 µm. Except for the antennal lobe region of the classical mPNs, the background clones in these images were masked as described in Material and Methods.

Besides PNs, the lAL lineage yields LNs. For most of the lineage, PNs and LNs were made in pairs, as the mitotic recombination during GMC divisions consistently led to the labeling of one PN paired with one LN by ts-MARCM (Figure 4). However, the final nine PN types were not paired with another neuron or a NB clone (Figure 4). This indicates that either the paired cell died or could not be labeled with *nSyb-GAL4*. Notably, the longer PN hemilineage exhibits higher morphological diversity than its LN sister hemilineage.

**Figure 5:**
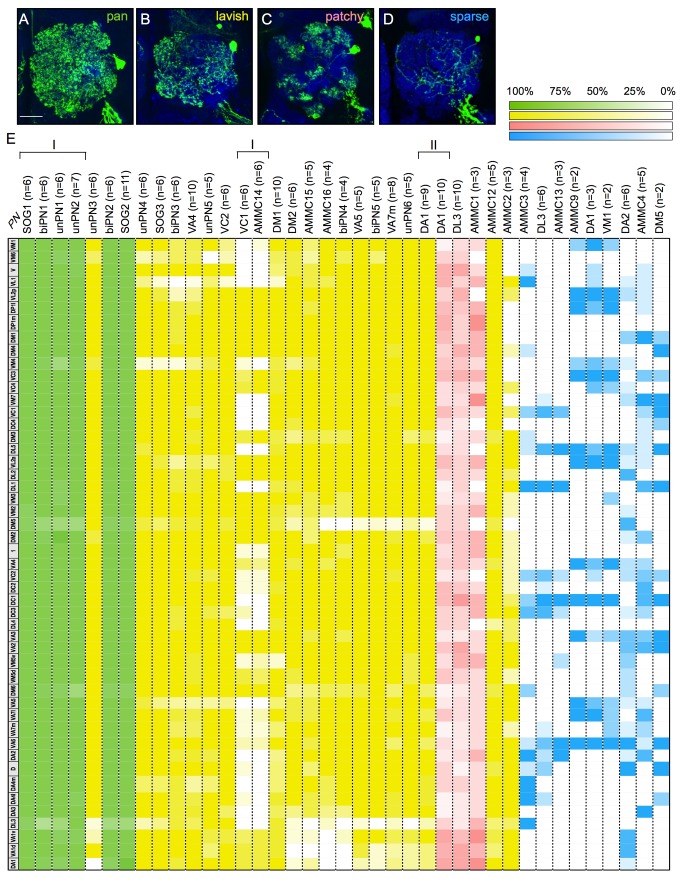
Annotation of lAL LNs based on birth order and neuron morphology. (A–D) Representative images of the four major classes of lAL LNs: pan-glomeruli (pan) (A), lavish (B), patchy (C), and sparse (D). The LNs (green) were labeled by ts-MARCM with *nSyb-GAL4*. Their PN sibs are not shown. Brains were counterstained with nc82 mAb (blue). Scale bar: 20 µm. (E) The table shows the glomeruli innervation patterns of the LNs paired with different PN types. Each row represents one glomerulus. Each column, separated by the dashed lines, represents the average glomerular innervation pattern for the LNs that are paired with the PN type shown on top (*n* indicates how many LNs were averaged). The LNs of different classes are labeled in different colors (pan, green; lavish, yellow; patchy, pink; sparse, blue). The chance for the LNs paired with the same PN type to innervate a particular glomerulus is color-coded as shown on the top-right corner of the figure. The LNs and their associated PNs are arranged by their birth order with early born on the left and later born on the right. Note the presence of several developmental windows where morphologically indistinguishable LNs can be associated with multiple sequentially produced PN types (two such examples are marked by “I” on the top of the table). A different window shows the association of one PN type (DA1 mPN) with two sequentially produced LN classes (marked by “II” on the top of the table).

First, unlike PNs that innervate brain regions involved in multiple sensory modalities, their paired LNs exclusively innervate the ALs and should be selectively involved in olfaction. Second, many distinct PNs were paired with indistinguishable LNs. Nonetheless, the LNs can be grouped into four classes based on the extent of their AL elaborations. The pan-AL LNs densely innervate all the glomeruli in the AL; the lavish LNs occupy most, but not all, AL glomeruli; the patchy LNs invade many glomeruli in spotty patterns; the sparse LNs, by contrast, arborize locally within a few glomeruli (Figure 5A–D). Notably, except for the DA1 and DL3 mPNs, PNs of a given type consistently pair with a particular class of LNs. The DA1 mPNs may be born with lavish, patchy, or sparse LNs, and the DL3 mPNs can pair with patchy or sparse LNs. By contrast, the remaining 43 PN types show strict sisterhood with one of the four LN classes. Taking both PN and LN diversities into consideration, we have in total recovered 48 distinct PN/LN pairs (Figure 4) that arise sequentially from the lAL lineage as implicated from the invariant birth order of the PNs (Figure 3A).

For those five PN/LN pairs (referred to as DA1/lavish, DA1/patchy, DA1/sparse, DL3/patchy, and DL3/sparse, respectively) whose distinction depends on the LN diversity, we refined the PN grouping and determined the subgroups' windows of production. We found that DA1/lavish, DA1/patchy, and DL3/patchy are born earlier in a contiguous sequence and that DL3/sparse and DA1/sparse are born later in separate windows (Figure 3A). When these 48 recognizable PN/LN pairs were chronologically arranged based on the derived birth order (Figure 4), we noticed that, unlike PNs, the AL LNs of different classes have arisen in a more logical sequence with most pan-AL LNs (Figure 4A–D,F,G) born before the lavish LNs (Figure 4E,H–Y, AC–AD), which largely precede the patchy LNs (Figure 4Z–AB) and ultimately transit to the sparse LNs (Figure 4AE–AM).

The pan-AL LNs paired with distinct PNs are morphologically indistinguishable from one another. They show analogous electrophysiological profiles [38], further indicating the homogeneity of the pan-AL class of LNs. How about the other three classes of LNs? Notably, the lavish or sparse LNs that associate with a particular PN type (thus born in a specific developmental time window) tend to avoid or innervate a characteristic set of AL glomeruli. To examine the LN diversity in further detail, we computed the average AL elaboration pattern of the LNs for each of the 48 sequentially derived PN/LN pairs. We manually annotated individual LNs' glomerular innervation patterns and then calculated the percentage of LNs, for a given PN/LN-pair type, whose neurites could be found within a particular glomerulus (Figure 5E). A uniform full pattern of elaboration was ascertained in the pan-AL LNs paired with distinct PNs (Figure 5). By contrast, the patchy LNs innervate various glomeruli stochastically and may jointly tile the entire AL, as they collectively show a low-penetrant targeting to nearly all the AL glomeruli within any of the three PN/LN groups that carry patchy LNs (Figure 5). Unlike the pan-AL and patchy LNs, the lavish as well as sparse LNs exhibit discriminative patterns of elaboration depending on the identity of the associated PNs. The lavish LNs selectively avoid certain glomeruli, while the sparse LNs preferentially innervate specific glomeruli (Figure 5). The stereotyped patterns of AL glomerular innervation observed in the LNs, paired with distinct PNs and born at specific developmental times, argue for the presence of distinct types of lavish and sparse LNs. This is distinct from the lack of discernible cellular diversity among the pan-AL or patchy LNs.

### The Projection Neuron and Local Interneuron Hemilineages Alter Temporal Identity Independently

Using the glomerular innervation frequencies to represent the LNs associated with a particular PN type and arranging them chronologically based on the deduced PN birth order revealed that the PN and LN hemilineages alter temporal identity independently. The PN hemilineage is longer and yields many more morphologically distinct neurons than the LN hemilineage. In addition, contrasting with PNs that arise in a rather complex sequence, the four LN classes are produced roughly in the order of pan-AL→lavish→patchy→sparse (Figures 4 and 5E). During the production of the relatively homogeneous pools of pan-AL or patchy LNs, we witnessed multiple unilateral temporal fate changes in the PN hemilineage (Figure 5E). As to the lavish and sparse LNs that exhibit morphological subtypes, we found that LNs showing indistinguishable AL elaboration patterns are born in contiguous blocks that yield distinct PNs (Figure 5E). These observations collectively indicate that LNs alter temporal identity (that controls morphogenesis) at a slower tempo than PNs do, although they are derived from the same GMCs. Despite the presence of fewer LN fate transitions, the lavish-to-patchy LN fate switch consistently occurs without a concomitant PN fate change. It subdivides the window of DA1 mPN neurogenesis into two blocks that differ only on the LN side (Figures 3A, 4Y–Z, and 5E). Taken together, PNs and LNs undergo independent temporal identity changes.

The independent PN/LN temporal fate specification is further evidenced by two unilateral PN fate duplications. The DA1 and DL3 mPNs were initially made at 46 to 58 h after larval hatching (ALH) paired with lavish or patch LNs. After that, the lAL NB switched to produce AMMC PNs paired with various LNs. Notably, around 70 to 84 h ALH, the lAL lineage yielded additional DL3 and DA1 mPNs in reverse birth-order and associated with sparse LNs (Figures 3A and 4Y,Z,AF,AI). These phenomena collectively suggest that neuronal terminal fates are determined in hemilineage-specific manners.

### Differential Notch Activity Governs the Differential Temporal Patterning Observed between Hemilineages

It is hard to image how the temporal fates of twin neurons can be differentially patterned, given that neuronal temporal identities are presumably conferred in the precursors by a set of sequentially and transiently expressed transcription factors [8],[22]. However, we have learned that the PN versus LN binary cell fates are determined through differential Notch signaling due to asymmetric segregation of Numb [24],[28]. We wondered if Notch merely specifies PN/LN binary fates or it also governs the differential patterning of PN and LN temporal fates. LNs were grossly transformed into PNs in the lAL NB clones that lacked Sanpodo (Spdo) (see below), a positive regulator of Notch [12],[39],[40]. Analyzing the temporal fates for those PNs transformed from LNs due to loss of Notch should help elucidate the role(s) of Notch in specifying PN versus LN temporal fates.

**Figure 6:**
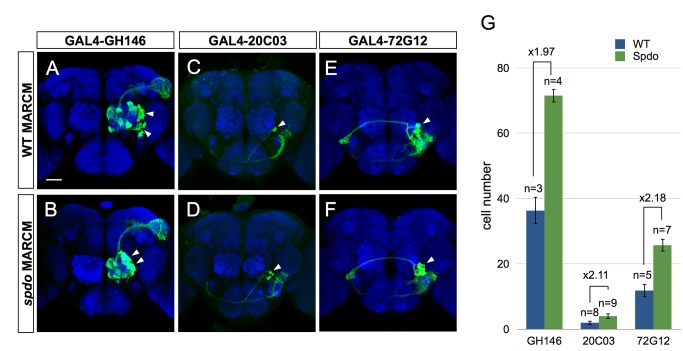
Complete duplication of the PN hemilineage in the absence of Spdo. (A–F) Wild-type (A,C,E) and *spdo* (B,D,F) MARCM NB clones induced at newly hatched larval stage. lAL clones were labeled by *GAL4-GH146* (A,B), *GAL4-20C03* (C,D), or *GAL4-72G12* (E,F). In all cases, the overall morphologies are the same between the wild-type and *spdo* mutant clones. The cell bodies are marked by arrowheads. Scale bar: 40 µm. (G) The bar graph shows the statistical result of the cell number of the lAL MARCM clones showing in (A–F).

We examined the PN composition of the PN-only *spdo* mutant lAL NB clones. We selectively marked the 12 types of mPNs, born in multiple clusters from 18 to 96 h ALH, with *GAL4-GH146*. We further checked distinct populations of AMMC neurons using *GAL4-GR20C03* and *GAL4-GR72G12*. We found that the *spdo* mutant lAL NB clones, labeled with any of the three GAL4 drivers, show wild-type morphologies but carry two times more cell bodies (Figure 6). These observations indicate a perfect duplication of the PN hemilineage in the *spdo* mutant clones and suggest that the transformed PN hemilineage undergoes the same temporal identity changes as the native PN hemilineage does. Such results argue that the differential Notch signaling not only promotes the LN or PN fate but also governs the differential manifestation of temporal identity changes in the LN versus PN hemilineage.

### Notch Specifies the AMMC Fates in the Notch-Low Projection Neuron Hemilineage through a *spdo*-Independent Pathway

**Figure 7:**
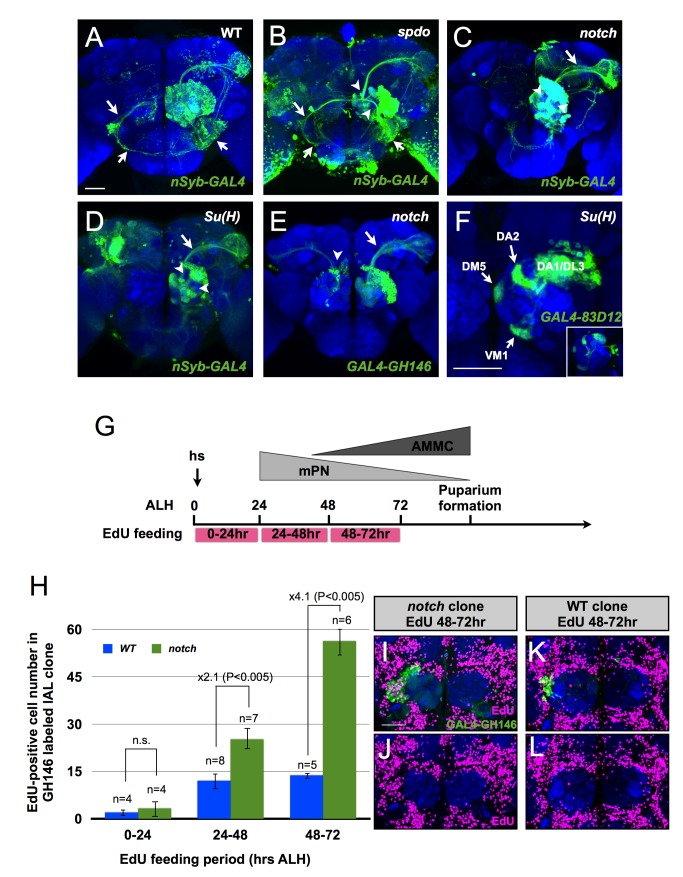
The specification of AMMC PNs requires a Spdo-independent Notch activity. (A–F) Wild-type (A), *spdo* (B), *notch* (C,E), and *Su(H)* (D,F) MARCM clones (green) labeled by *nSyb-GAL4* (A–D), *GAL4-GH146* (E), and *GAL4-83D12* (F). Brains were counterstained with nc82 mAb (blue). The arrows in (A) and (B) indicate the neurites of the AMMC PNs. The arrowheads in (B–D) indicate several regions in the antennal lobe not covered by the clones. The arrow in (C–E) indicates the antennocerebral (iACT) projection mainly from the GH146-positive lAL mPNs. The arrowhead in (E) indicates a background clone of the anterodorsal projection neuron lineage. The insect in (F) shows a wild-type *GAL4-83D12* clone. Scale bar: 40 µm. (G–J) Wild-Type (WT) and *notch* MARCM clones labeled by *GAL4-GH146* were induced at 0–2 h after larval hatching (ALH). After clone induction, the larvae were fed EdU for 24 h at 0–24, 24–48, or 48–72 h ALH. The EdU-positive cell numbers in the clones were counted at the adult stage. (G) The illustration shows the normal developmental windows for mPNs and AMMC PNs, the time of heat-shock (hs) for inducing MARCM clones, and the EdU-feeding periods along the larval development. (H) The bar graph shows the number of the EdU-positive cells (*y*-axis) in the *GAL4-GH146*-labeled WT (blue) and *notch* (green) MARCM clones in the flies fed with EdU at 0–24, 24–48, and 48–72 h ALH (*x*-axis). The error bars are standard deviation. The *p* value was calculated using Student *t* test. (I–L) Examples of *notch*- (green in I) or wild-type-clone (green in K)–containing adult brains from the flies fed with EdU at 48–72 h ALH. The brain was counterstained with anti-EdU (magenta) and nc82 (blue) Abs. The images in (J,L) are the same as that in (I,K) but show only the EdU staining. Note comparable numbers of EdU-positive cells on the lateral side of the ALs between the left and right hemispheres. Scale bar: 40 µm.

In contrast with the faithful duplication of diverse PNs in the *spdo* mutant clones, the lAL NB clones homozygous for mutations in *notch* or its co-activator *Su(H)* exhibited abnormal PN compositions. Labeling entire clones with *nSyb-GAL4* revealed missing of the AMMC neurite tracks specifically in the *notch* or *Su(H)* mutant NB clones (Figure 7C,D, compared to Figure 7A,B). There was no evidence for cell loss, given that we consistently counted around 200 cell bodies regardless of the clone genotype. To exclude changes in the pattern of lAL neurogenesis, we further determined the rate of proliferation at 30 h ALH when the lAL NB mainly produces mPNs and at 70 h ALH when the AMMC neurons are made. The sizes of wild-type and *Su(H)* clones were comparable. Moreover, they carried analogous numbers of mitotic cells (Table S2) as revealed with the mitosis marker phospho-histone H3 (PH3) [41]. So the PN-only *notch* and *Su(H)* mutant clones have made GMCs that yield viable neurons in correct numbers and at right timings, making us wonder if the prospective AMMC neurons have adopted other PN fates and acquired non-AMMC neurite trajectories. Given the prominence of AL neuronal elaborations in those clones lacking AMMC trajectories, we examined if the *notch* and *Su(H)* mutant NB clones carry many more AL PNs at the expense of AMMC neurons. We found that *notch* mutant lAL NB clones contain about five times more GH146-positive AL neurons than wild-type controls (Figure 7E, compared to Figure 6A). A three times increase in the numbers of the later-born DA1, DL3, VM1, DA2, and DM5 mPNs, visualized with *GAL4-GR83D12*, was also observed in Su*(H)* mutant clones (Figure 7F). Note the exclusive dense innervation of the DA1, DL3, VM1, DA2, and DM5 glomeruli by the much enlarged *Su(H)* mutant clones (Figure 7F), indicating an excessive production of normal-looking AL PNs by the lAL NB deficit in *notch* or *Su(H)*. These observations suggest that the prospective AMMC neurons of *notch*/*Su(H)* mutant clones might have aberrantly adopted the AL PN fates characteristic of siblings born at different times, reminiscent of some temporal cell fate transformation.

The majority of AMMC neurons are born after 60 h ALH (Figure 7G). If the prospective AMMC neurons had been transformed into AL PNs, one would expect that the supernumerary AL PNs were largely added during the second half of the lAL lineage. To verify this viewpoint, we examined when the GH146-positive mPNs were made in excess by the *notch* mutant lAL NB. We fed the larvae harboring *GAL4-GH146*-labeled wild-type or *notch* clones with EdU, a thymidine analog that labels proliferating cells, for 1 d at 0–24, 24–48, or 48–72 h ALH (Figure 7G). The pulse labeling of EdU first confirmed that the GH146-positive mPNs were mostly generated between 24 and 72 h ALH (Figure 7H). It further revealed that the majority of the excessive GH146-positive neurons in the *notch* mutant lAL NB clones were born after 48 h ALH when the prospective AMMC neurons were supposed to arise. Compared to wild-type controls, *notch* mutant clones yielded two times more GH146-positive neurons at 24–48 h ALH and up to four times more at 48–72 h ALH (Figure 7H). This increase was not due to an acceleration of NB proliferation, because the total numbers of the EdU-positive cells on the lateral side of the AL remained comparable to those of the wild-type controls (Figure 7I–L). And the 4-fold increase at 48–72 h ALH cannot be fully accounted for by the LN-to-PN fate changes. It argues instead that, on top of the binary cell fate transformation, most, if not all, of the PNs yielded during that period, including those that normally adopt the AMMC neuronal fates, have uniformly developed into GH146-positive mPNs.

In sum, Notch signaling underlies the specification of AMMC versus AL neurons in the Notch-low PN hemilineage. Interestingly, the positive regulator of Notch, Spdo, is essential for the binary cell fate decision between LNs and PNs but dispensable for the temporal fate specification of the AMMC versus AL PN fates.

### Analogous Dynamic Chinmo Expression Governs PN and LN Temporal Fates in Hemilineage-Dependent Manners

**Figure 8:**
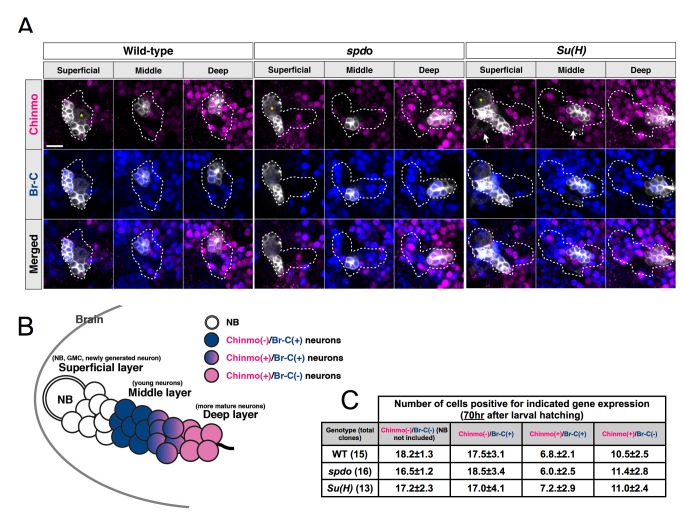
The temporal transition from Chinmo to Br-C is not affected by loss of Notch signaling. (A) The *asense-GAL4*-labeled wild-type, *spdo*, and *Su(H)* lAL clones (white) immunostained for Chinmo (magenta) and Br-C (blue). Single confocal planes at superficial, middle, and deep layers are shown. The white signals indicated by arrows are background clones. Scale bar: 10 µm. (B) The illustration shows the composition of the lAL lineage at 70 h ALH based on the Chinmo and Br-C antibody staining. At the superficial layer, the NB, GMCs, and newly generated neurons are negative for both Chinmo and Br-C. At the middle layer, the young neurons are positive for Br-C but negative for Chinmo. The neurons located slightly deeper are positive for Chinmo and Br-C, and the earlier born neurons in the deep layer are only positive for Chinmo. (C) The table shows the numbers of cells positive for Chinmo, Br-C, or both in *asense-GAL4*-labeled wild-type (WT), *spdo*, and *Su(H)* lAL clones at 70 h ALH.

Notch might regulate neuronal temporal cell fates through refining temporal codes or modulating postmitotic neurons' responses to Notch-independent transcriptional cascades. Chinmo and Br-C are dynamically expressed during larval neurogenesis [30],[31]. We wondered if such dynamic gene expressions exist in the developing lAL lineage and whether these temporal signatures vary depending on Notch activities. Consistent with previous reports [31], we could reliably detect a sequential birth-order-dependent expression of Chinmo and Br-C in the neuronal offspring of most, if not all, larval brain NBs. Chinmo preceded Br-C in the partially overlapping temporal gene expression, such that Chinmo(+)/Br-C(−) neurons consistently reside deeper in the cell body layer than their Chinmo(−)/Br-C(+) siblings (Figure 8A,B). We quantified the lAL offspring positive for Chinmo and/or Br-C at 70 h ALH when many AMMC precursors should already exist. We obtained comparable numbers of Chinmo(+)/Br-C(−), Chinmo(+)/Br-C(+), and Chinmo(−)/Br-C(+) neurons in the lAL NB clones regardless of the genotype of *spdo* or *Su(H)* (Figure 8C). We conclude that the Chinmo→Br-C temporal expression takes place analogously in both PN and LN hemilineages and independently of Notch activities.

**Figure 9:**
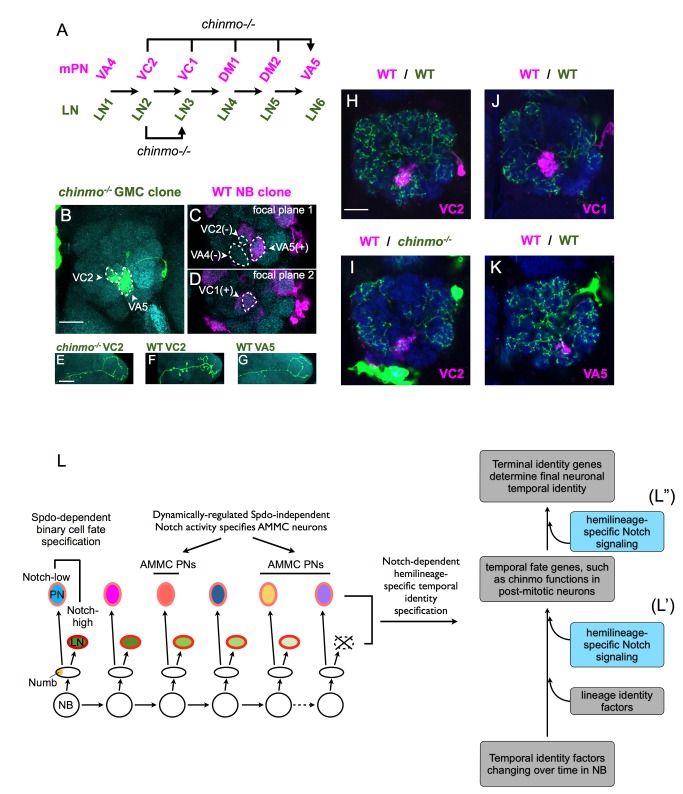
The PN and the LN hemilineages respond to *chinmo* loss-of-function differently. (A) The illustration summarizes the PN and LN temporal fate changes due to loss of *chinmo* function. LN1–6 represent the LNs that are normally paired with the corresponding mPNs shown on top. Without *chinmo*, the cells that normally differentiate into VC2 mPNs adopt the much later VA5 mPN fate, whereas the cells that normally differentiate into the LN2 aberrantly acquire the next temporal fate. (B–D) A *GAL4-GH146*-labeled ts-MARCM clone that marks a *chinmo* ^−/−^ VC2 mPN in green (A) and the rest of the lineage in magenta (C,D). The brain was counterstained with anti-nc82 Ab (cyan). The *chinmo* ^−/−^ VC2 mPN mainly innervates VA5 glomerulus with residual elaboration in VC2 glomerulus (B). The rest of the lineage contains only the neurons born after VC2 mPN, such as VA5 mPN (C) and VC1 mPN (D), but not the neurons born before VC2 mPN, such as VA4 mPN (C). Scale bar: 20 µm. (E–G) ts-MARCM clones of a *chinmo* ^−/−^ VC2 mPN (E), a WT VC2 mPN (F), and a WT VA5 mPN (G). Only the single-PN side of the ts-MARCM clones and only axon projections in the lateral horn are shown. Note the *chinmo* ^−/−^ VC2 mPN is more similar to the WT VA5 mPN than the WT VC2 mPN. Scale bar: 20 µm. (H–K) The *nSyb-GAL4* labeled ts-MARCM two-cell clones of a wild-type (WT) VC2/WT LN2 pair (H), a WT VC2/*chinmo^−/−^* LN2 pair (I), a WT VC1/WT LN3 pair (J), and a WT VA5/WT LN6 pair (K). The PNs and LNs were pseudocolored in magenta and green, respectively. The brains were counterstained with anti-nc82 Ab (blue). Note the *chinmo* ^−/−^ LN2 (I) is more similar to the WT LN3 (J) than the WT LN2 (H) and the WT LN6 (K). Scale bar: 20 µm. (L–L″) A model illustrating how neuronal diversity is regulated by Notch in the lAL lineage. The lAL neuroblast (NB) undergoes repeated self-renewal divisions to yield a series of ganglion mother cells (GMCs). Each GMC divides asymmetrically to generate a Notch-high and a Notch-low sister cells. The differential Notch activity specifies one cell as a local interneuron (LN; Notch-high) and the other as a projection neuron (PN; Notch-low) through a Spdo-dependent pathway, except at the end of the lineage where the Notch-high cells die before adulthood. In the Notch-low PN hemilineage, Notch activity is dynamically regulated by an unknown mechanism and promotes AMMC neuron fates through a Spdo-independent pathway. Furthermore, the hemilineage-specific Notch activity also regulates hemilineage-specific temporal identity specification by two potential mechanisms (L′,L″). The lAL NB sequentially expresses a series of temporal identity factors (TIFs), analogous to Hunchback→Krupple→Pdm→Caster observed during embryonic neurogenesis [22]. The lAL GMCs inherit the transiently expressed TIFs. The TIFs work together with lineage identity factors to turn on downstream temporal fate genes such as *chinmo* in the postmitotic neurons. Notch might act with lineage-specific factors and TIFs to regulate the expression of temporal fate genes other than *chinmo* (L′). Alternatively, differential Notch signaling could govern the derivation of terminal identity genes in hemilineage-specific manners through the control of postmitotic neurons' responses to common temporal fate genes (L″).

We further examined the involvement of Chinmo in specifying neuronal temporal fates of PNs versus LNs. Using *GAL4-GH146* to monitor the orderly production of the 12 types of mPNs with ts-MARCM, we demonstrated the requirement of Chinmo for proper specification of the VC2, VC1, DM1, and DM2 temporal fates (Figure 9A). All of them have aberrantly adopted the VA5 temporal fate following loss of Chinmo from respective GMCs, as evidenced by their targeting of the VA5 glomerulus and the branching of axons reminiscent of the wild-type VA5 mPNs (Figure 9B–G for VC2; unpublished data for VC1, DM1, and DM2). We then examined Chinmo's requirement for their twin LNs. We created mutant LNs paired with wild-type PNs as isolated two-cell clones. Based on AL elaboration patterns, the *chinmo* mutant LN of the VC2 mPNs (LN2) has adopted the fate of LN3 (the twin LN of the VC1 mPN) rather than the fate of LN6 (the twin LN of the VA5 mPN) (Figure 9A). Compared to the wild-type LN2 innervating near all AL glomeruli (Figure 9H), the prospective LN2 homozygous for *chinmo* acquired a much more restricted pattern of neurite elaboration and resembled the next-born LN3 (Figure 9I,J). The transformed LN2 appears distinct from LN6 that normally pairs with the VA5 mPN (Figure 9K), although the *chinmo* ^−/−^ VC2 mPN has consistently adopted the VA5 mPN fate (Figure 9A–G). We did not observe *chinmo*-related temporal identity phenotypes for other LNs examined so far.

Taken together, we identified *chinmo* as a temporal fating factor in the lAL lineage. Notably, the Notch-independent dynamic expression of Chinmo governs LN and PN temporal fates in hemilineage-specific (i.e., Notch-dependent) manners, arguing that Notch acts in parallel with or downstream of temporal fating factors to determine terminal temporal fates (Figure 9L).

## Discussion

Detailed analysis of the lateral antennal lobe (lAL) lineage attests to the stereotypy of clonal development in the *Drosophila* central brain, discloses novel types of antennal lobe (AL) neurons as well as neurons that innervate other brain regions, and exemplifies how diverse neurons of different classes can derive from a common progenitor. The lAL neuroblast (NB) gives rise to a rather heterogeneous population of neurons, which is achieved through the derivation of two distinct hemilineages that yield projection neurons (PNs) and local interneurons (LNs), respectively. The LN hemilineage produces LNs exclusively for the AL, while the PN hemilineage generates not only AL PNs but also PNs of the antennal mechanosensory and motor center (AMMC) and suboesophageal ganglion (SOG). Furthermore, the paired hemilineages yield diverse PNs and LNs concurrently but in distinct temporal patterns. Various neurons of different LN classes are made one class after another. By contrast, distinct PNs arise in a complex intercalated sequence. Given that most *Drosophila* neuronal lineages (possibly all except the MB lineages) consist of two distinct hemilineages or exist as a lone hemilineage [25],[26], neural development and neuronal diversification appear to be orchestrated along hemilineages instead. This suggests that understanding hemilineage identity will clarify a central organizational theme in *Drosophila* brain development.

Interestingly, Notch governs hemilineage identity and further patterns the hemilineage-characteristic temporal fate changes. By lineage mapping using ts-MARCM and through analysis of *sanpodo* (*spdo*) mutant clones, we confirm that the lAL lineage is made up of a Notch-high LN hemilineage and a Notch-low PN hemilineage. Despite their derivation from common GMCs, PNs and LNs undergo temporal fate changes independently. The lAL PNs exhibit higher cellular diversity and thus alter their temporal fates more frequently than their LN sibs do. However, there are also developmental periods when one LN interclass fate switch consistently occurs during the continuous production of a particular PN type. Notably, knocking down *spdo* through the lAL lineage development has resulted in duplication of the entire PN hemilineage. This indicates that the prospective LNs have been transformed into PNs with correct PN temporal fates. It argues that twin neurons are born with identical temporal fating factors and that the Spdo-dependent Notch activity has not only promoted the LN fate but also governed the birth time/order-dependent neuronal diversification in the LN hemilineage.

And Notch mediates cell fate decision between LNs and PNs as well as within the Notch-low PN hemilineage where Notch acts in a Spdo-dispensable manner to promote the AMMC PN fates as opposed to the AL PN fates (Figure 9L). In the PN-only *Notch* or *Su(H)*, but not *spdo*, mutant lAL NB clones, the prospective AMMC neurons aberrantly adopted various AL PN fates. Diverse AMMC neurons and distinct AL PNs normally arise in alternative blocks. Regardless of the AMMC-to-AL fate transformation, the overall temporal patterning appeared intact in the AMMC-lacking PN hemilineages as evidenced by comparable increases in the AL PNs of various types (Figure 7). These observations suggest that Notch is not involved in the regulation of GMC temporal identity but rather diversifies PN temporal fates after birth of postmitotic neurons. At this stage, we are still naïve about the nature of such Spdo-independent Notch signaling or the sources of the dynamics that underlie the alternation of AMMC and AL PN fates.

Two mechanisms could underlie the Notch-dependent temporal fate specification of both PNs and LNs (Figure 9L′,L″). First, Notch High or Low may differentially modulate the refinement of temporal fating factors in the newborn neurons. Lineage identity genes have been shown to participate in subpatterning of temporal cell fates in the NB 5–6 lineage [23]. It is possible that terminal identity genes are established in postmitotic neurons through a combined action of lineage determinants, GMCs' temporal identity factors and Notch signaling (Figure 9L′). Second, Notch targets may modulate neuronal responses to common temporal codes. Notably, the birth-order-dependent expressions of Chinmo and Br-C in the lAL offspring were well maintained even when loss of Notch signaling had elicited complex binary and temporal fate transformations. And the Notch-independent dynamic expression of Chinmo governed both PN and LN temporal fates but in hemilineage-specific manners. These observations imply that Notch acts downstream of temporal fating factors to regulate neuronal temporal fates potentially through some epigenetic mechanisms (Figure 9L″).

As to the neuronal details and their possible functions, the lAL lineage yields diverse classes of AL LNs and PNs, many distinct AMMC neurons, and a small number of SOG PNs, which may contribute to the processing of various sensory inputs. Beside the 12 types of well-characterized monoglomerular PNs (mPNs) that connect a single glomerulus of the AL to mushroom body (MB) calyx and lateral horn (LH) [34],[35], we identified eight types of unilateral PN (unPN) and six types of bilateral PN (biPN). The unPNs have proximal elaboration in the ipsilateral AL and biPNs have that in both ipsilateral and contralateral AL. Interestingly, unPNs and biPNs often connect AL to brain regions that have not been shown to be involved in olfaction, such as posteriorlateral protocerebrum (PLP), superior medial protocerebrum (SMP), inferior ventrolateral protocerebrum (IVLP), and crepine (CRE) (Figure S1). In addition to these putative olfactory neurons, there are 16 types of AMMC PNs and three types of SOG PNs in the lAL lineage. The SOG PNs have proximal innervation in suboesophageal ganglion (SOG), the primary target for the gustatory receptor neurons [42], and therefore are candidate downstream neurons in the gustatory processing neural circuit. The AMMC PNs have primary innervations in the antennal mechanosensory and motor center (AMMC), which have been shown to be important for hearing and gravity-sensing [43]. The AMMC PNs therefore might be part of the auditory/gravity-sensing circuit [43],[44]. Notably, like many AL PNs, most AMMC PNs have axon-like projection into IVLP (Figure S1). Such convergence makes IVLP a potential integration site for various inputs. The production of diverse PNs from a single progenitor further suggests a possible evolution of distinct networks from a common ancestral circuit.

In sum, the lAL NB makes multiple classes of diverse neurons in a complex yet stereotyped pattern, manifested as a series of LN/PN pairs and orchestrated through distinct Notch activities. The Spdo-dependent Notch action that occurs in the Numb-negative offspring has not only conferred the LN fate but also patterned the LN temporal identities. A novel Spdo-independent Notch action is further utilized to increase the PN temporal fates by promoting AMMC neuronal fates in otherwise AL PNs. Both Notch-mediated temporal fate regulations are apparently executed after proper deployment of temporal fating factors. Taken together, Notch plays integral roles in the derivation of final neuronal temporal cell fates.

## Materials and Methods

### Fly Strains

The fly strains used in this study include (1) *GAL4-GH146* [45]; (2) *asense-GAL4*; (3) *FRT19A,notch[55e11],UAS-mCD8::GFP*; (4) *FRT40A,UAS-mCD8::GFP,UAS-rCD2i,Chinmo[1],GAL4-GH146/CyO*; (5) *hs-FLP[1];FRT40A,UAS-rCD2::RFP,UAS-GFPi*; (6) *FRT40A,UAS-mCD8::GFP,UAS-rCD2i;nSyb-GAL4(2-1)*; (7) *FRT82B,spdo[27]/TM6B*; (8) *40A,Su(H)[delta* 47*]*/*CyO* [46],[47]; (9) *FRT19A,hs-FLP[122],tubp-GAL80;GAL4-GH146*; (10) *FRT19A,hs-FLP[1];nSyb-GAL4*; (11) *hs-FLP[1];GAL4-GH146;FRT82B,tubp-GAL80*; (12) *hs-FLP[1];FRT82B,UAS-rCD2::RFP-UAS-GFPi*; and (13) *FRT82B,spdo[27],UAS-mCD8::GFP-UAS-rCD2i*.

### MARCM and ts-MARCM Clonal Analysis

Larvae 0–2 h old with proper genotype were collected and put into vials (80 larvae/vial) containing standard fly food. The larvae were raised at 25°C until desired stages. To induce clones, the larvae were heat-shocked at 37°C for 15–40 min. After heat shock, the larvae were put back to 25°C until dissection at desired stages. Only male flies were dissected for the detailed lineage analysis of the lAL neurons. Because background olfactory receptor neuron (ORN) clones often interfered with the lAL clones in the antennal lobe, we removed antennae 1 d after adult eclosion and waited for 3 d for the ORN axons to degenerate before brain dissection. For the ts-MARCM clones in Figures 1B, 2, and 4, the clones of interest inevitably coexist with various background clones due to the use of the pan-neuronal driver *nSyb-GAL4*. In such cases, confocal images of the brains containing clones of the same neuron type were carefully compared stack by stack to determine the background clones. The brain with the least background was chosen and the background clones were manually masked to reveal the clone of interest.

### EdU Incorporation Assay

Larvae 0–2 h old with the genotype of *FRT19A,notch[1],UAS-mCD8::GFP/hs-FLP[122],FRT19A,tubp-GAL80;GAL4-GH146,UAS-mCD8::GFP/CyO* or *FRT19A,UAS-mCD8::GFP/hs-FLP[122],FRT19A,tubp-GAL80;GAL4-GH146,UAS-mCD8::GFP/CyO* were heat-shocked at 37°C for 1 h to induce MARCM clones. To feed the larvae EdU at 0–24 h ALH, the larvae were transferred into vials (100 larvae/vial) containing standard fly food with 100 µg/ml EdU (Invitrogen) for 24 h at 25°C, and then transferred into vials (100 larvae/vial) with standard fly food only until adult eclosion. To feed the larvae EdU at 24–48 h or 48–72 h ALH, the larvae, after heat-shock, were transferred into vials (100 larvae/vial) containing standard fly food for 24 h or 48 h at 25°C. The larvae were then transferred into vials (100 larvae/vial) containing standard fly food with 100 µg/ml EdU for 24 h at 25°C. After the EdU feeding, the larvae were transferred back to the vials with standard fly food and raised at 25°C until adult eclosion. The adult brains were dissected in 1× Phosphate buffered saline (PBS) and stained for EdU using Click-iT EdU Alexa Fluor 555 Imaging Kit (Invitrogen). After the EdU staining, the brains were washed three times by 1× PBS with 0.75% Triton X-100 (0.75% PBT; Fisher Scientific) for 15 min each. The brains were then incubated with rabbit anti-GFP Ab (1∶1,000; invitrogen) and mouse nc82 mAb (1∶50; DSHB) at 4°C overnight. Next day, the brains were washed with 0.75% PBT three times for 15 min each and incubated with Alexa 488-conjugated goat anti-rabbit (1∶200; invitrogen) and Cy5-conjugated goat anti-mouse secondary antibodies (1∶400; Jackson ImmunoResearch) at 4°C overnight. Next day, the brains were washed with 0.75% PBT three times for 15 min each before mounted using SlowFade gold anti-fade reagent (Invitrogen).

### Immunohistochemistry and Microscopy

Fly brains were dissected in 1× PBS, fixed in 1× PBS with 4% formaldehyde (Fisher Scientific) at room temperature for 20 min, washed by 1× PBS with 0.75% Triton X-100 (0.75% PBT; Fisher Scientific) three times for 15 min each, and incubated in 1× PBS with 0.5% goat normal serum (Jackson ImmunoResearch) for 30 min before incubation with primary antibodies at 4°C overnight. Next day, the brains were washed in 0.75% PBT three times for 15 min each before incubated with secondary antibodies at 4°C overnight. Next day, the brains were washed with 0.75% PBT for 15 min for three times and mounted using SlowFade gold anti-fade reagent (Invitrogen). The immunofluorescent signals were collected by Zeiss LSM confocal microscope and processed using Fiji and Adobe Photoshop. Primary antibodies used in this study include rat anti-mCD8 mAb (1∶100; Caltag), mouse nc82 mAb (1∶100; DSHB), rabbit anti-Dsred (1∶500; Clontech), mouse anti-Br-C (core) (1∶100; DSHB), rabbit anti-Chinmo (1∶1,000) [30], rabbit anti-PH3 (1∶250; Upstate), and rabbit anti-GFP Ab (1∶1,000; invitrogen). The secondary antibodies were Alexa 488-conjugated goat anti-rabbit or goat anti-rat (1∶200; invitrogen), Cy3-conjugated goat anti-rabbit (1∶400; Jackson ImmunoResearch), and Cy5-conjugated goat anti-mouse (1∶400; Jackson ImmunoResearch).

## Supporting Information

Figure S1 The projectome of lAL PNs. Schematic illustration of lAL PNs (arrows) and their projections among various brain regions. The directions of arrows indicate putative information flow from dendrites to axonal terminals. The mPNs, unPNs, and biPNs (labeled in green) have dendritic arborization in the AL, probably involved in olfactory circuit (green). The AMMC PNs (labeled in blue) possibly contribute to the auditory/gravity-sensing neural network (blue). The SOG PNs (labeled in gray) are likely involved in the gustatory neural circuit (gray). The PLP receives many inputs from the AL and thus might be another odor information-processing center besides the MB calyx (Ca) and lateral horn (LH). In addition, the IVLP receives inputs from AL as well as AMMC and potentially integrates olfactory and auditory/gravity information. AL, antennal lobe; cAL, contralateral AL; PLP, posteriorlateral protocerebrum; PVLP, posterior ventrolateral protocerebrum; cPVLP, contralateral posterior ventrolateral protocerebrum; SOG, suboesophageal ganglion; IB, inferior bridge; Ca, mushroom body calyx; LH, lateral horn; AMMC, antennal mechanosensory and motor center; cAMMC, contralateral antennal mechanosensory and motor center; IVLP, inferior ventrolateral protocerebrum; cIVLP, contralateral inferior ventrolateral protocerebrum; SMP, superior medial protocerebrum; CRE, crepine.
(TIF)

Table S1 The description of the lateral antennal lobe neurons. The different zones in AMMC and their corresponding sensory inputs were identified based on Kamikouchi et al. [48].
(TIF)

Table S2 The average number of PH3-positive cells and percentage of PH3-positive neuroblasts (NBs) in wild-type (WT), *spdo*, and *Su(H)* lAL clones at 30 h and 70 h ALH.
(TIF)

## Abbreviations

ACdT: antennocerebral descending tract
adPN: anterodorsal projection neuron
AL: antennal lobe
ALH: after larval hatching
AMMC: antennal mechanosensory and motor center
biPN: bilateral projection neuron
Ca: mushroom body calyx
cAL: contralateral antennal lobe
cAMMC: contralateral antennal mechanosensory and motor center
cIVLP: contralateral inferior ventrolateral protocerebrum
CNS: central nervous system
cPVLP: contralateral posterior ventrolateral protocerebrum
CRE: crepine
GMC: ganglion mother cell
iACT: inner antennalcerebral tract
iAMMCc: inferior AMMC commissure
IB: inferior bridge
IVLP: inferior ventrolateral protocerebrum
lAL: lateral antennal lobe
LH: lateral horn
LN: local interneuron
MARCM: mosaic analysis with a repressible cell marker
MB: mushroom body
mPN: monoglomerulus projection neuron
NB: neuroblast
oACT: outer antennal-cerebral tract
PLP: posteriorlateral protocerebrum
PN: projection neuron
PVLP: posterior ventrolateral protocerebrum
sAMMCc: superior AMMC commissure
SMP: superior medial protocerebrum
SOG: suboesophageal ganglion
ts-MARCM: twin-spot mosaic analysis with repressible cell markers
unPN: unilateral projection neuron
vPN: ventral projection neuron
