# Supplementary figures and images for: Lineage Analysis of Drosophila Lateral Antennal Lobe Neurons Reveals Notch-Dependent Binary Temporal Fate Decisions
Source: PLoS Biol. 2012 Nov 20;10(11):e1001425. doi: 10.1371/journal.pbio.1001425 (PMC3502534; doi:10.1371/journal.pbio.1001425)

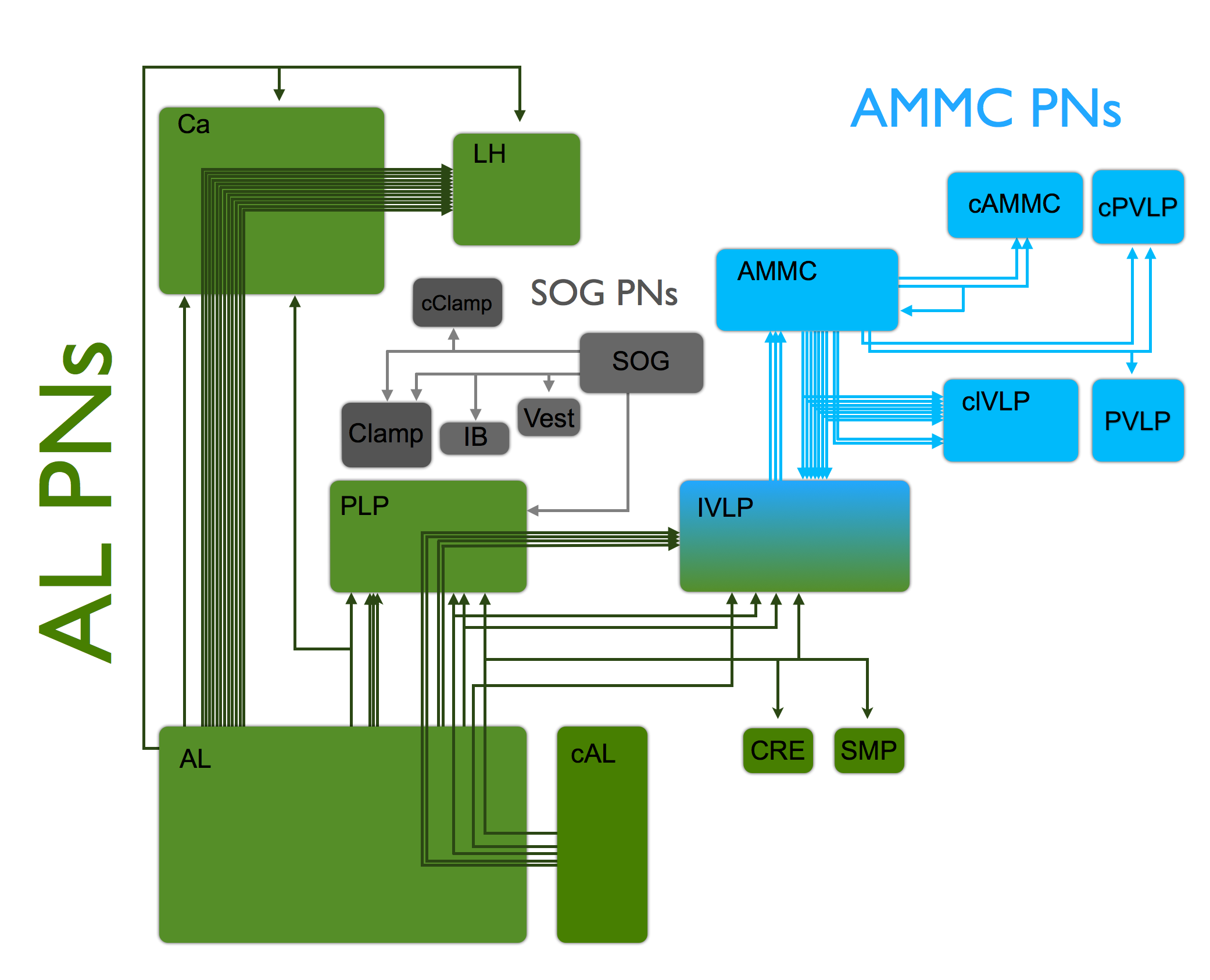

Supplement: Figure S1 — The projectome of lAL PNs. Schematic illustration of lAL PNs (arrows) and their projections among various brain regions. The directions of arrows indicate putative information flow from dendrites to axonal terminals. The mPNs, unPNs, and biPNs (labeled in green) have dendritic arborization in the AL, probably involved in olfactory circuit (green). The AMMC PNs (labeled in blue) possibly contribute to the auditory/gravity-sensing neural network (blue). The SOG PNs (labeled in gray) are likely involved in the gustatory neural circuit (gray). The PLP receives many inputs from the AL and thus might be another odor information-processing center besides the MB calyx (Ca) and lateral horn (LH). In addition, the IVLP receives inputs from AL as well as AMMC and potentially integrates olfactory and auditory/gravity information. AL, antennal lobe; cAL, contralateral AL; PLP, posteriorlateral protocerebrum; PVLP, posterior ventrolateral protocerebrum; cPVLP, contralateral posterior ventrolateral protocerebrum; SOG, suboesophageal ganglion; IB, inferior bridge; Ca, mushroom body calyx; LH, lateral horn; AMMC, antennal mechanosensory and motor center; cAMMC, contralateral antennal mechanosensory and motor center; IVLP, inferior ventrolateral protocerebrum; cIVLP, contralateral inferior ventrolateral protocerebrum; SMP, superior medial protocerebrum; CRE, crepine. (TIF) [file pbio.1001425.s001.tif]

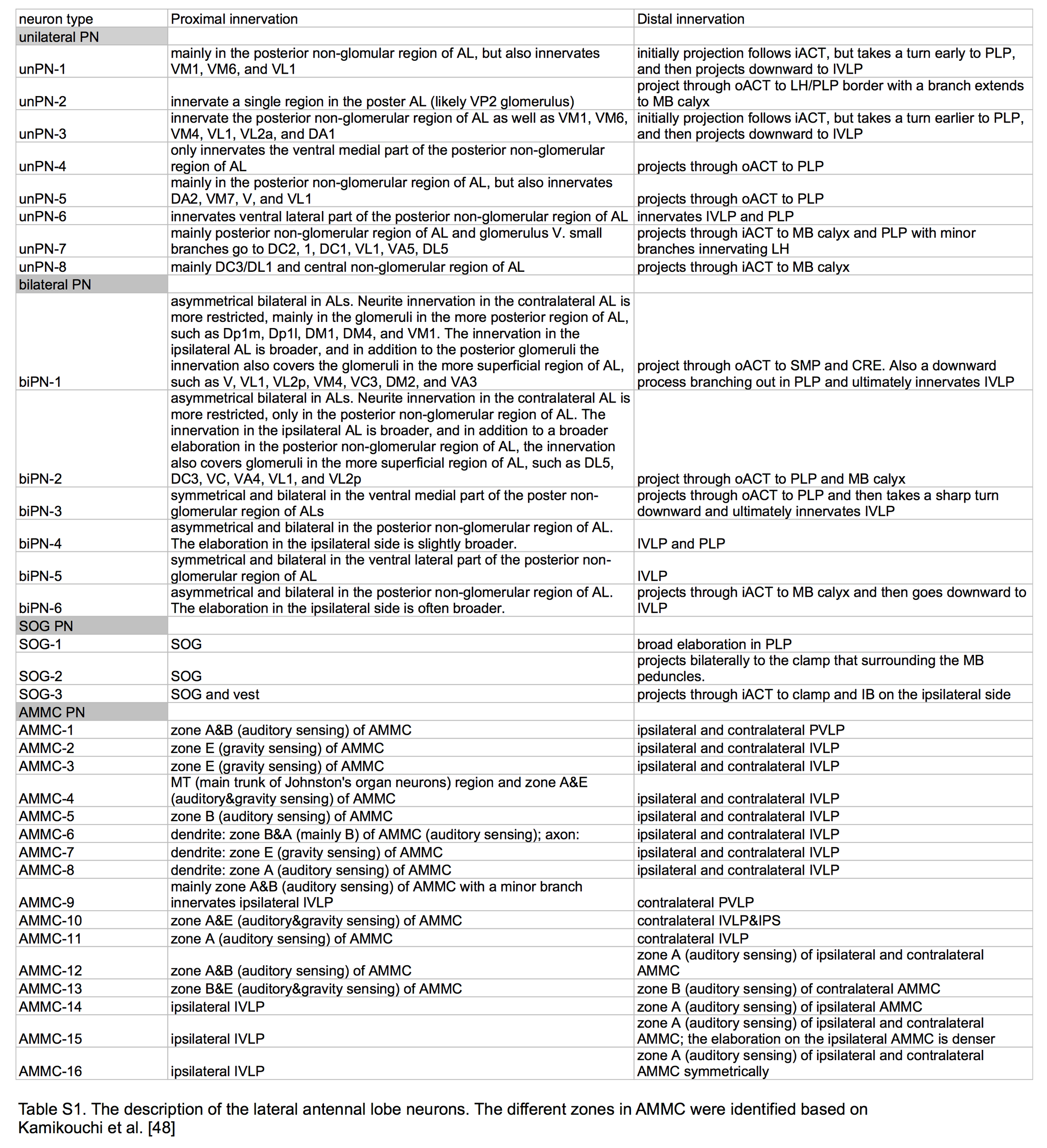

Supplement: Table S1 — The description of the lateral antennal lobe neurons. The different zones in AMMC and their corresponding sensory inputs were identified based on Kamikouchi et al. [48]. (TIF) [file pbio.1001425.s002.tif]

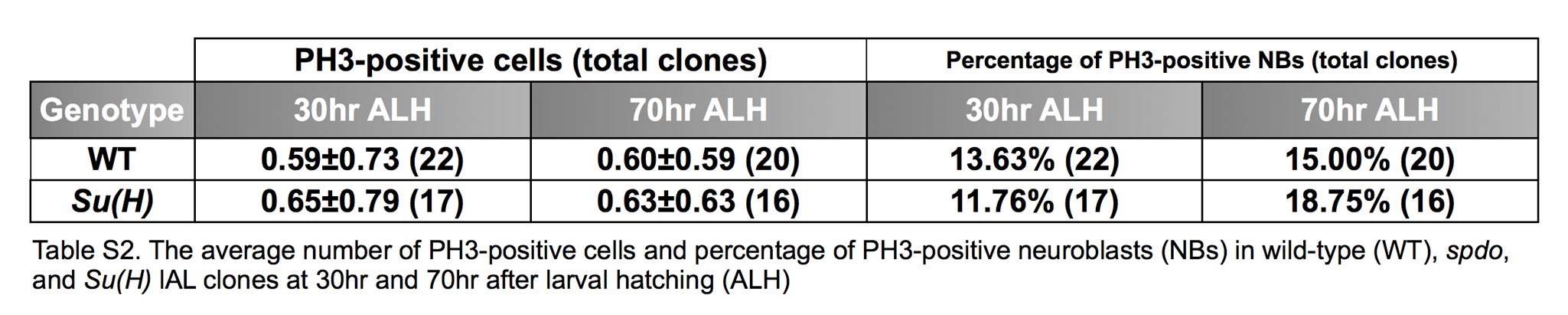

Supplement: Table S2 — The average number of PH3-positive cells and percentage of PH3-positive neuroblasts (NBs) in wild-type (WT), spdo, and Su(H) lAL clones at 30 h and 70 h ALH. (TIF) [file pbio.1001425.s003.tif]
